# *Phytophthora infestans* Has a Plethora of Phospholipase D Enzymes Including a Subclass That Has Extracellular Activity

**DOI:** 10.1371/journal.pone.0017767

**Published:** 2011-03-14

**Authors:** Harold J. G. Meijer, Hussen Harrun Hassen, Francine Govers

**Affiliations:** 1 Laboratory of Phytopathology, Plant Sciences Group, Wageningen University, Wageningen, The Netherlands; 2 Centre for BioSystems Genomics (CBSG), Wageningen, The Netherlands; J. Craig Venter Institute, United States of America

## Abstract

In eukaryotes phospholipase D (PLD) is involved in many cellular processes. Currently little is known about PLDs in oomycetes. Here we report that the oomycete plant pathogen *Phytophthora infestans* has a large repertoire of PLDs divided over six subfamilies: PXPH-PLD, PXTM-PLD, TM-PLD, PLD-likes, and type A and B sPLD-likes. Since the latter have signal peptides we developed a method using metabolically labelled phospholipids to monitor if *P. infestans* secretes PLD. In extracellular medium of ten *P. infestans* strains PLD activity was detected as demonstrated by the production of phosphatidic acid and the PLD specific marker phosphatidylalcohol.

## Introduction

In eukaryotes, phospholipid-based signalling and metabolism play important roles in numerous cellular processes. Phospholipids are ubiquitous components of all cell membranes and, as second messengers, they act as modulators of many cellular functions. Conversion of phospholipids is accomplished by enzymes that are well conserved across eukaryotes and play crucial roles in cellular regulation, metabolism, stress responses and phospholipid biosynthesis. Important classes of phospholipid modifying enzymes are phospholipid kinases, phospholipid phosphatases and phospholipases. The latter are a heterogeneous group of enzymes whose classification is based on their catalytic activity and substrate specificity.

Phospholipase D (PLD) catalyzes the hydrolysis of structural phospholipids at their terminal phosphoesteric bond, leading to the production of a hydrophilic constituent and phosphatidic acid (PA). The latter has emerged as a significant lipid mediator in many cellular processes. PA mediated signal transduction includes modulation of receptor signalling, cytoskeleton rearrangement, secretion, and vesicle trafficking during endocytosis or exocytosis [1]. All eukaryotes have PXPH-PLDs that are composed of the N-terminally located phosphoinositide binding domains Pleckstrin homology (PH) and PHOX homology (PX). These precede the catalytic site and regulatory motifs. Unique to plants are the C2-PLDs that contain the N-terminally located calcium/lipid binding domain C2. These two PLD subfamilies have a catalytic site with two highly conserved motifs each consisting of HxKxxxxD (hereafter HKD1 and HKD2). Bacteria have PLDs that harbour the catalytic site and regulatory motifs but lack phosphoinositide binding domains. Also “non-HKD-PLDs” have been identified, enzymes with PLD activity but lacking characteristic motifs [2]. Recently, a novel rice (*Oryza sativa*) PLD was described that contains the two HKD motifs as well as a N-terminal signal peptide (SP) [3].

Annotation of the genomes of *Phytophthora sojae* and *Phytophthora ramorum* revealed that *Phytophthora* has a variable set of PLD genes. *Phytophthora* lacks C2-PLD genes but possesses one PXPH-PLD gene. In addition, there are seventeen PLD genes that represent novel subfamilies including a subfamily with SP sequences that encodes potentially secreted PLDs [4]. In this study we performed an in depth analysis of the PLD subfamilies in *Phytophthora infestans,* the causal agent of potato late blight and the most notorious *Phytophthora* species. All *Phytophthora* PLD subfamilies were detected but the subfamily of PLDs with SPs (sPLD-likes) appears to be comprised of two unrelated subfamilies, sPLD-like-A and sPLD-like–B. Moreover, we developed a method that makes use of reconstituted plant membrane vesicles for analysing the presence of PLD activity in extracellular medium of *P. infestans* and provide evidence that *P. infestans* indeed secretes PLD. This finding points to a putative role for PLD in pathogenicity.

## Materials and Methods

### Bioinformatic analysis

Putative PLD genes in the *P. infestans* genome database of strain T30-4 (http://www.broad.mit.edu/annotation/genome/phytophthora_infestans) were identified by several methods including automatic annotation, BLAST searches with the *P. sojae* and *P. ramorum* PLD gene models [4], and with representative PLD sequences from NCBI GenBank. Putative PLD gene models were further analysed based on characteristic conserved motifs and manually corrected when needed. Multiple alignments were made in ClustallW2. Phylograms were constructed by Mega4.1 using the Minimum Evolution method with a bootstrap test based on 5000 replicates, and the Poisson correction method [5]. Signal and anchor peptides were detected via SignalP 3.0 (http://www.cbs.dtu.dk/services/SignalP/) using default parameters. Putative transmembrane domains were analysed as described before [6]. Searches for additional proteins domains were performed via publically available databases. For determining synteny between *Phytophthora* spp. gene models present in regions flanking PLD genes were extracted and used for reciprocal BLAST analysis. Pairs of genes sharing best reciprocal BLAST hits were assigned as orthologues. Intergenic lengths were determined by taking the distance between the PLD gene and the neighbouring gene model thereby ignoring mobile elements.

### *Phytophthora infestans* culture condition and sampling

*P. infestans* strains were routinely grown at 18°C in the dark on V8 media or on Rye agar medium supplemented with 2% sucrose [7]. Extracellular medium was obtained by flooding full grown plates with V8 medium. After overnight incubation samples were taken and immediately centrifuged for 2 min at 10,000 g. The supernatant was collected and filtered over 0.2 µM filters to remove cell debris.

### Plant cell suspension culturing and radio-labelling of phospholipids

Cell suspensions of *Nicotiana tabacum* were grown as described [8]. Metabolically labelled ^32^P-labelled phospholipids were obtained by overnight incubation of 1–2 ml cell suspension with 100 µCi carrier-free ^32^PO_4_^3−^ (GE Healthcare, Diegem, Belgium). The labelled cells were divided into 200 µl aliquots in 2-ml Eppendorf vials and labelling was terminated by the addition of 20 µl perchloric acid (50%, v/v). After brief vortexing, the samples were frozen in liquid nitrogen. After 5 min, 450 µl CHCl_3_:MeOH:HCl (50∶100∶1, v/v) was added and the mixture was briefly mixed and snap-frozen in liquid nitrogen. During thawing the tubes were shaken vigorously and thereafter snap-frozen again to improve lipid recovery. After subsequent thawing, 450 µl CHCl_3_ and 200 µl 0.9% (w/v) NaCl was added to induce a two-phase system. Tubes were then vigorously shaken for 15–30 min and centrifuged for 2 min. The lower-phase was transferred to a fresh tube containing 450 µl CHCl_3_:MeOH:1 M HCl (3∶48∶47, v/v), mixed for 1 min and centrifuged for 2 min. Subsequently the upper-phase was removed and the lower organic phase was dried under a stream of N_2_(g) and the isolated lipids were dissolved in 20 µl CHCl_3_ and stored at −20°C until usage.

### Phospholipase D assay

The metabolically labelled phospholipids were dried under N_2_(g) and resuspended into vesicles in 200 µl 10 mM Tris buffer (pH 6.4) by sonication (VWR ultrasonic cleaner). Aliquots (10 µl) were supplemented with 5 µl buffer containing 40% propanol. The assay was initiated by adding 85 µl medium (control) or medium in which *P. infestans* was cultured (extracellular medium), followed by a 1 hour incubation at room-temperature. The reaction was stopped by adding 375 µl CHCl_3_:MeOH:HCl (50∶100∶1 [v/v]), and processed as described [8].

### TLC analysis

Lipid samples were separated by thin layer chromatography on Merck silica 60 TLC plates (Darmstadt, Germany) using the ethyl acetate system as described [9]. Radiolabelled phospholipids were visualized by phosphoimaging (Storm, Molecular Dynamics; Sunnyvale, CA, USA).

## Results

### Sixteen genes in *P. infestans* encode proteins with phospholipase D hallmarks

The genome sequence of *P. infestans* harbours eighteen genes encoding a PLD, all of which can be classified in the subfamilies identified previously in *P. sojae* and *P. ramorum* (Table 1). PXPH-PLD, PXTM-PLD and TM-PLD are single copy genes. Three genes encode members of the PLD-like subfamily and the remaining twelve encode putatively secreted PLDs (sPLD-likes) based on the presence of a predicted SP. Alignments revealed that eleven sPLD-likes share sustained protein similarity (>50% overall identity) whereas one (sPLD-like-1) lacks significant identity with any of the others (15%; Table S1). sPLD-like-1 was therefore designated as a type A (sPLD-like-A) and the others as type B (sPLD-like-B). Two of the latter, sPLD-like-4 and -11, lack continuous open reading frames (ORFs) and are considered as pseudogenes. In retrospect, also *P. sojae* and *P. ramorum* have one sPLD-like-A ortholog (Table 1).

**Table 1:** PLDs in Phytophthora infestans.

Class	Proposed name	Gene number	Scaffold	Protein accession	Intron	Protein length	Signal peptide prediction	ESTs Nr.	*P. sojae* homolog (BlastP)	*P. ramorum* homolog (BlastP)	Highest homology, BlastP hit (acc. nr., organism, E-value); Sequences outside *P. infestans* strain T30-4
PXPH-PLD	PXPH-PLD	PITG_03651	4	EEY66109	1	1119	-	5	PLD_134882	PLD_84787	CBI22957; *Vitis vinifera*; 2e-141
PXTM-PLD	PXTM-PLD	PITG_00284	1	EEY57717	0	1807	-	3	PLD_163010	PLD_101442	Q5BMR2; *Phytophthora infestans*; 0.0
TM-PLD	TM-PLD	PITG_16798	49	EEY65487	0	874	-	2	PLD_128805	PLD_81941	XP_002452125; *Sorghum bicolor*; 1e-12
sPLD-like-A	sPLD-like-1	PITG_18185	63	EEY67671	0	549	0.484	2	PLD_139486	PLD_81884	XP_002515425; *Ricinus communis*; 5e-55
sPLD-like-B	sPLD-like-2	PITG_00616	1	EEY58009	0	558	0.998	0	PLD_140963	PLD_79972	YP_003339462; *Streptosporangium roseum*; 1e-33
sPLD-like-B	sPLD-like-3	PITG_20602	181	EEY56690	0	558	0.997	0	PLD_140963	PLD_79972	YP_003339462; *Streptosporangium roseu*m; 4e-31
sPLD-like-B	sPLD-like-4*	PITG_20603	181	EEY56691	0	562	1	0	PLD_140963	PLD_79972	YP_003383471; *Kribbella flavida; 6e-36*
sPLD-like-B	sPLD-like-5	PITG_00617	1	EEY58010	0	562	1	0	PLD_140963	PLD_72867	YP_003383471; *Kribbella flavida; 2e-36*
sPLD-like-B	sPLD-like-6	PITG_22809	10	EEY53326	0	568	1	0	PLD_163070	PLD_101467	YP_003383471; *Kribbella flavida; 9e-38*
sPLD-like-B	sPLD-like-7	PITG_06994	10	EEY53348	0	571	0.991	0	PLD_138732	PLD_101476	YP_001106428; *Saccharopolyspora erythraea*; 2e-36
sPLD-like-B	sPLD-like-8	PITG_10568	18	EEY57013	0	578	0.997	0	PLD_138544	PLD_84378	YP_003339462; *Streptosporangium roseum*; 1e-38
sPLD-like-B	sPLD-like-9	PITG_10572	18	EEY57017	0	589	1	0	PLD_138537	PLD_72867	YP_884788; *Mycobacterium smegmatis;* 2e-40
sPLD-like-B	sPLD-like-10	PITG_10563	18	EEY57009	0	598	0.999	0	PLD_138537	PLD_101476	YP_884788; *Mycobacterium smegmatis;* 2e-37
sPLD-like-B	sPLD-like-11*	PITG_12809	27	EEY60394	0	598	0.992	0	PLD_138732	PLD_72867	YP_884788; *Mycobacterium smegmatis*; 8e-36
sPLD-like-B	sPLD-like-12	PITG_12806	27	EEY60392	0	605	0.989	1	PLD_138537	PLD_101476	YP_884788; *Mycobacterium smegmatis*; 4e-39
PLD-like	PLD-like-1	PITG_00921	1	EEY58277	0	531	-	48	PLD_127024	PLD_77742	YP_003339462; *Streptosporangium roseum*; 2e-38
PLD-like	PLD-like-2	PITG_21129	228	EEY59066	0	542	-	0	PLD_138537	PLD_72867	YP_884788; *Mycobacterium smegmatis*; 1e-40
PLD-like	PLD-like-3	PITG_00923	1	EEY58278	0	556	-	0	PLD_127026	PLD_77744	YP_003383471; *Kribbella flavida;* 2e-37

*Pseudogenes; for the phylogram shown in Figure 2, the ORFs were ‘restored’ by introducing a ‘T’ at position 180741 in supercontig 1.181 for sPLD-like-4 and ‘GA’ at position 1472900 in supercontig 1.27.

The PLD proteins were analyzed for characteristic conserved motifs (Table 2). The two catalytic motifs HKD1 and HKD2 are conserved in *P. infestans* PXTM-PLD, PXPH-PLD and sPLD-like-A. In TM-PLD, the PLD-likes and sPLD-like-Bs one HKD motif is modified. In TM-PLD HKD2 is changed in HND, as in the *P. sojae* and *P. ramorum* TM-PLDs. The PLD-likes and sPLD-likes lack the aspartate residue (D) of HKD1 at the expected position.

**Table 2:** Conserved motifs detected in *P. infestans* PLDs.

				Conserved regulatory domains/motifs			
Name	Gene number	HKD1	HKD2	PIP_2_-binding	“IYIENQFF”	“DRY/RVYVVV”	“IGSANIN”
PXPH-PLD	PITG_03651	HKD	HKD	+	HFVYIENQFF	+(EKF)/+	IGSANIN
PXTM-PLD	PITG_00284	HKD	HKD	+	HFLYIENQFF	+(EKF)/+	LGSANIN
TM-PLD	PITG_16798	HKD	HKN	−	−	+(EPF)/−	−
sPLD-like-1	PITG_18185	HKD	HKD	−	−	−/−	*VGSANMD* *
sPLD-like-2	PITG_00616	HKL	HKD	+	NFIFIEDQYF	−/+	VGSANWN
sPLD-like-3	PITG_20602	HKL	HKD	+	NFIFIEDQYF	−/+	VGSANWN
sPLD-like-5	PITG_00617	HKT	HKD	+	NFIYIEDQYF	−/+	VGSANWN
sPLD-like-6	PITG_22809	HKT	HKD	+	NFIYVEDQYF	−/+	IGSANWN
sPLD-like-7	PITG_06994	HKA	HKD	+	NYIYIEDQYF	−/+	VGSSNWN
sPLD-like-8	PITG_10568	HKA	HKD	+	NFVYIEDQYF	−/+	DGSANWN
sPLD-like-9	PITG_10572	HKA	HKD	+	NFIYIEDQYF	−/+	VGSANWN
sPLD-like-10	PITG_10563	HKA	HKD	+	NFIYIEDQYF	−/+	LGSANWN
sPLD-like-12	PITG_12806	HKA	HKD	+	NFICIEDQYF	−/+	VGSANWN
PLD-like-1	PITG_00921	HKR	HKD	+	NYIFIQDQYF	−/+	LGSANWN
PLD-like-2	PITG_21129	HKA	HKD	+	NFIYIEDQYF	−/+	VGSANWN
PLD-like-3	PITG_00923	HKR	HKD	+	NYIFIQDQYF	−/+	LGSANWN
PXPH-PLD	PITG_03651	HKD	HKD	+	HFVYIENQFF	+(EKF)/+	IGSANIN

PIP_2_ binding domain and DRY/RVYVVV motifs are indicated as present (+), or absent (−). For other motifs the amino acid sequences are shown.

*This “IGSANIN” motif is found directly downstream of HKD1 (see text).

Additional motifs in PLDs have been recognized as regulatory or binding domains. In PXPH-PLD both the PX and PH domain are readily conserved. PXTM-PLD contains a PX domain and five transmembrane domains [6]. A remnant of a PH domain (E–value 1.64E^+03^) was detected between transmembrane domains two and three. As described previously PXTM-PLD has a “PIP_2_-binding domain”, a “PC-binding site” (IYIENQFF motif) and a Gα-protein binding motif DRY/RVYVVV between HKD1 and HKD2 [6]. In human HsPLD2 and *Arabidopsis* AtPLDδ, the PIP_2_-binding domain was recently identified as a tubulin binding region and the IYIENQFF and the DRY/VYVVV motifs appear to act as an actin binding fragment [10], [11], [12]. *P. infestans* PXPH-PLD and PXTM-PLD encompass all these domains. Both, the PIP_2_-binding domain and the IYIENQFF motif are present in PLD-likes and sPLD-like-Bs. Notably, in these cases the C-terminal hydrophobic region of the DRY/RVYVVV motif is conserved but the DRY motif is not found. In PXTM-PLD and plant PLDs it is replaced by EKF [6]. In TM-PLD an EPF is located at the expected position but the hydrophobic region is lacking. sPLD-like-1 solely consists of the catalytic site with the two HKD motifs (Table 2).

Downstream of HKD2, PXPH-PLDs and C2-PLDs contain a so-called IGSANIN motif that is supposed to play a role in membrane attachment. The motif is fully conserved in PXPH-PLD whereas in PXTM-PLD, the PLD-likes and sPLD-like-Bs a slightly modified motif is found. In TM-PLD the motif is barely recognizable; only two out of seven amino acids are conserved. sPLD-like-A lacks the motif but instead has a “VGSANMD” motif that is located directly downstream of HKD1 (Table 2).

### Phylogenetic relationships of PLD genes

**Figure 1:**
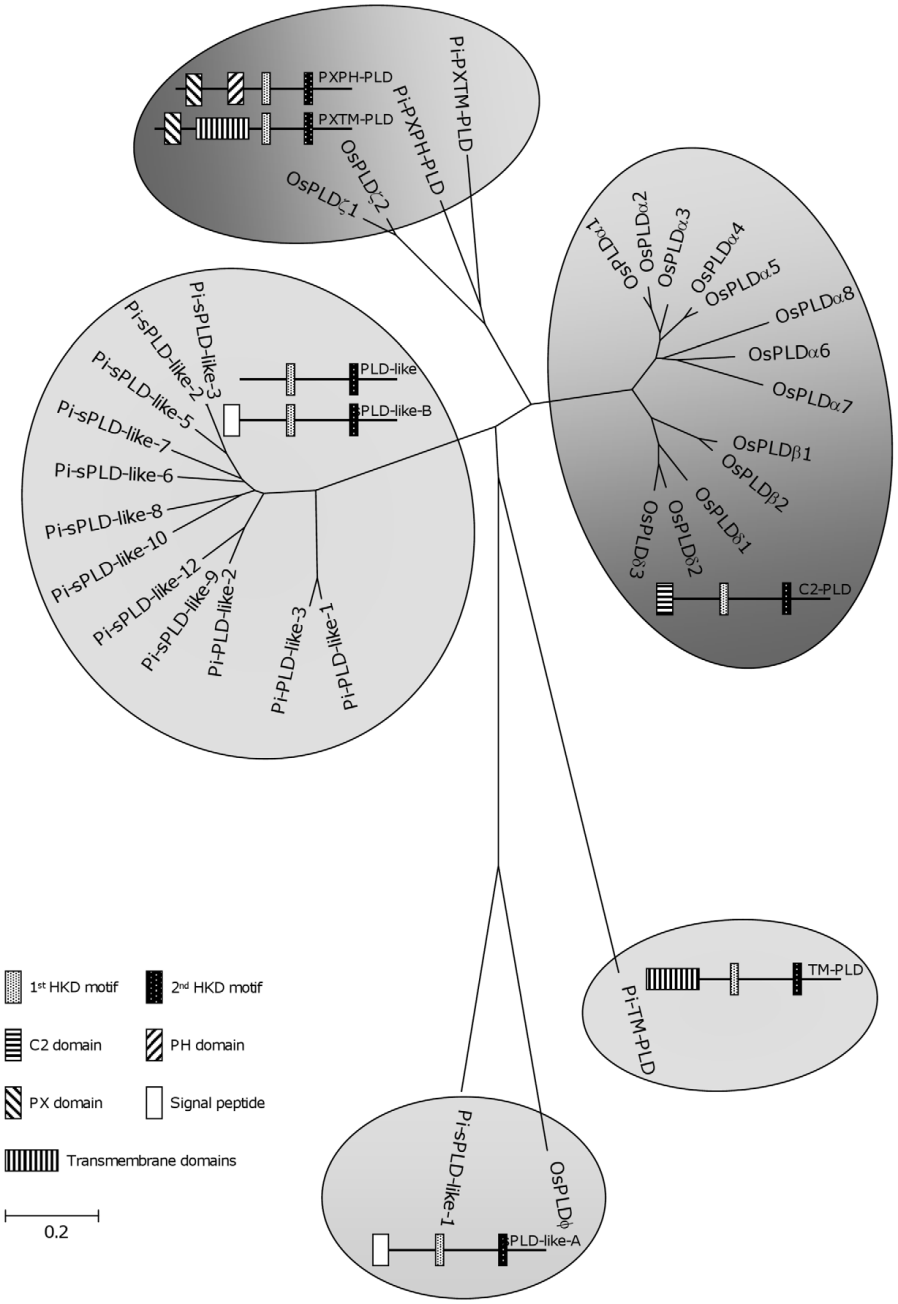
Phylogram of PLDs in *P. infestans* and rice (*Oryza sativa*). A consensus minimal evolution tree was constructed based on the AA sequences comprising the catalytic motifs and intermediate regions of PLDs in *P. infestans* and *O. sativa*. OsPLDκ was excluded from the analysis because it lacks the catalytic motifs. For accession numbers of the *P. infestans* sequences see Table 1. For rice sequences see Li et al. [3].

To analyse the relationship between *Phytophthora* PLDs and those of other organisms, PLD sequences were retrieved from genome databases. For an overall comparison, *P. infestans* PLDs were clustered with the 16 PLDs encoded in the rice genome. Rice has a putatively secreted PLD (OsPLDϕ];) and hence, has three PLD subfamilies [3].*P. infestans* PXPH-PLD and PXTM-PLD cluster with rice PXPH-PLDs. Most sPLD-likes and PLD-likes cluster in one large clade separated from the rice C2-PLDs. The sPLD-like-A of *P. infestans* clusters with OsPLDφ. TM-PLD is an outlier that is absent in rice (Figure 1).

Further study revealed that PXPH-PLDs from plants and mammals are the closest homologs of *P. infestans* PXTM-PLD and PXPH-PLD (not shown). For TM-PLD, low similarity was found only with three *P. infestans* proteins with a DUF803 domain (PITG_05621, PITG_09535 and PITG_12469). Since the DUF803 domain corresponds to the transmembrane domains an additional analysis was performed for the C-terminal part harbouring the catalytic site. With an E-value of 6e^−6^ a PLD from *Phaeobacter gallaeciensis* (ZP_02144336) was the closest homolog.

### Evolution of (s)PLD-like genes in *Phytophthora*

**Figure 2:**
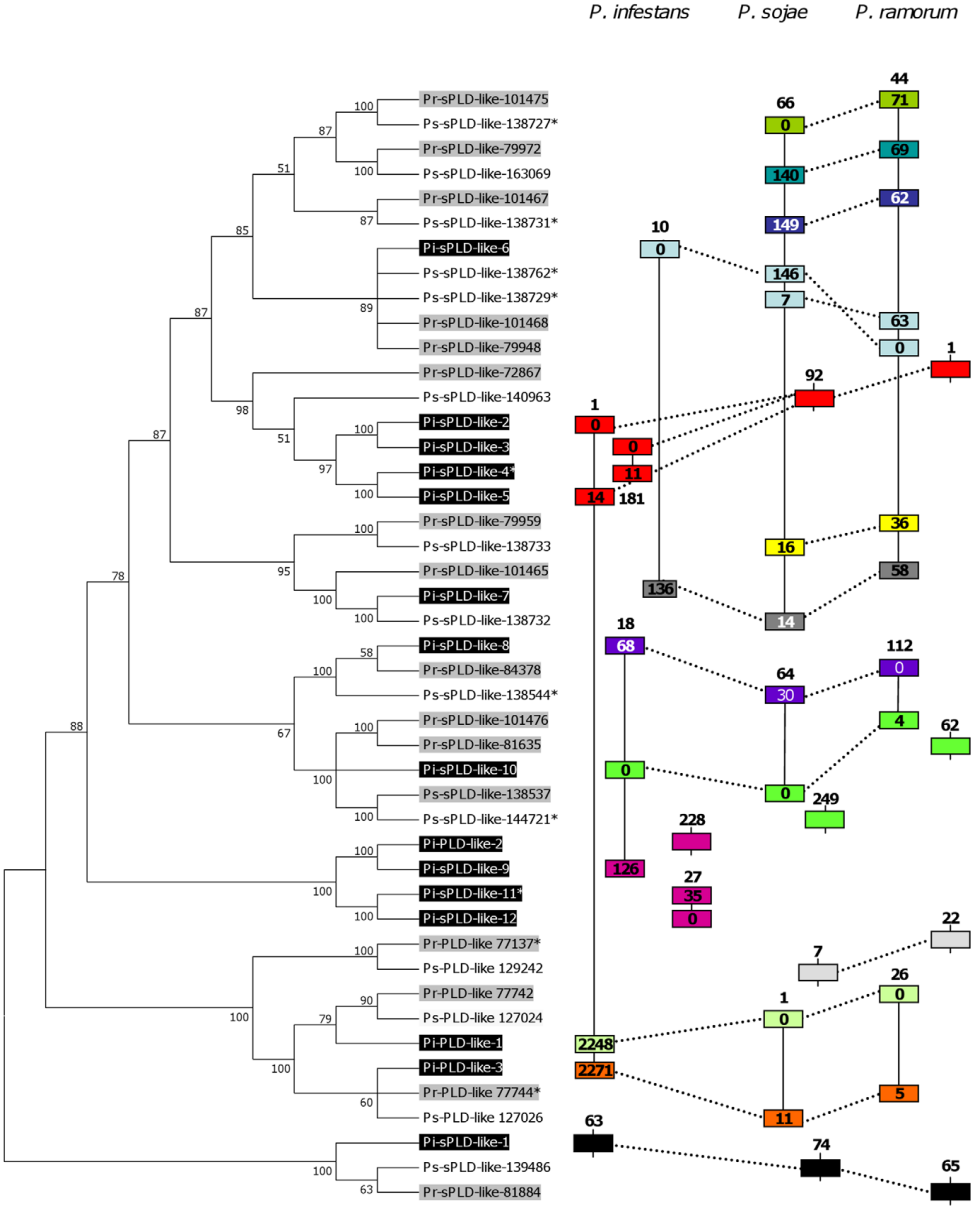
Phylogram and genome organization of *Phytophthora* PLD-likes and sPLD-likes. The phylogram shown on the left was constructed based on AA sequences. (s)PLD likes from *P. infestans* and *P. ramorum* are shown in black and gray respectively. Pseudogenes (marked by *; see Table 1 and [4]) were included in the analysis. On the right, the vertical lines represent scaffolds containing one or more (s)PLD-like genes (rectangles). The scaffold numbers are shown above or below each line. On scaffolds with two or more (s)PLD-like genes the number in each rectangle indicates the distance in kb between the start of this gene and the start of the first PLD-like gene (marked by 0) on the scaffold. (s)PLD-likes that cluster in the phylogram have the same color. Dotted lines connecting the scaffolds indicate that the genomic regions are syntenic.

The large number of PLD-like and sPLD-likes genes in *Phytophthora* could point to gene duplication events in a common ancestor. To investigate this we constructed a phylogram of all (s)PLD-likes in the three *Phytophthora* species (Figure 2). The PLD-likes with the exception of Pi-PLD-like-2 group in one clade and Pi-PLD-like-1 and -3 each have their own ortholog in *P. sojae* and *P. ramorum.* The bulk of the tree represents the sPLD-like-B family and remarkably includes Pi-PLD-like-2 that groups with three Pi-sPLD-like-B genes, i.e. Pi-sPLD-like-9, -11 and -12, in a distinct *P. infestans* specific subclade. Another *P. infestans* specific cluster is observed in the subclade comprising Pi-sPLD-like-2, -3, -4 and -5 which all four group with the same ortholog in *P. sojae* and *P. ramorum.* Other subclades lack a *P. infestans* ortholog. This non-uniform distribution is also evident in Table 1 in which the closest homologs of the *P. infestans* (s)PLD-like genes are listed based on BlastP. Several sPLD-like-Bs share the same *P. sojae* or *P. ramorum* protein as closest homolog rather than having unique sets of three orthologs representing one copy in each of the species. This suggests that some sPLD-like-Bs in *P. infestans* evolved directionally so that the paralogs are more related to each other than to orthologs. Despite this close relationship the paralogs still show significant differences at identity level (Table S1).

### *P. infestans* PLD genes are dispersed in the genome

The PLD genes in *P. infestans* are dispersed over nine scaffolds (Table 1; Figure 2). Five genes are located on scaffold_1, including two sPLD-like-B genes that are 12 kb apart, and two PLD-like genes, 21 kb apart. Three sPLD-like-B genes are located on scaffold_18 spanning 127 kb. Also the two sPLD-like-B genes on scaffold_10 are far apart (135 kb). Scaffold_27 and scaffold_181 have two sPLD-like-B genes whereas sPLD-like-A is located on scaffold_63. When compared to *P. sojae* and *P. ramorum*, the distribution of PLD genes within *P. infestans* seems more dispersed. For example, in *P. sojae*, six sPLD-like-B genes are located within an 18 kb region and similarly, in *P. ramorum*, five within 15 kb [4].

To examine the extent of synteny between species, genomic regions flanking each PLD gene were analysed. The average intergenic length was 1.7 kb, 2.4 kb and 6.7 kb, respectively, for *P. ramorum*, *P. sojae* and *P. infestans.* The regions carrying sPLD-like-1 (Figure 2) and the single copy genes PXPH-PLD, PXTM-PLD and TM-PLD (not shown) showed conserved synteny. The majority of the PLD-like and sPLD-like genes in *P. infestans* are surrounded by genes that co-localize with PLD-like and sPLD-like genes in the other two species although genes are often rearranged (Figure 2). Scaffold_181 of *P. infestans* might have been the result of a duplication event involving Scaffold_1. For both PLD-like-2 and sPLD-like-9, some of the surrounding genes were found on *P. sojae* scaffold_64 and *P. ramorum* scaffold_112 suggesting another duplication event. Alternatively, scaffold_228 which is only 184 kb in size, might in reality be part of scaffold_18. For genes flanking sPLD-like-11 and -12 homologs were found in the other two species but not in regions harbouring sPLD-like-B genes. Based on the phylogeny and the synteny it seems plausible that the two *P. infestans* specific sPLD-like-B clades arose by gene duplication after emergence of the species *infestans.* Moreover, since *P. sojae* and *P. ramorum* are more at the base of the *Phytophthora* phylogeny than *P. infestans* the absence of *P. infestans* orthologs in some subclades is probably due to gene loss.

### sPLD-like-A represents an independent peak in PLD evolution

**Figure 3:**
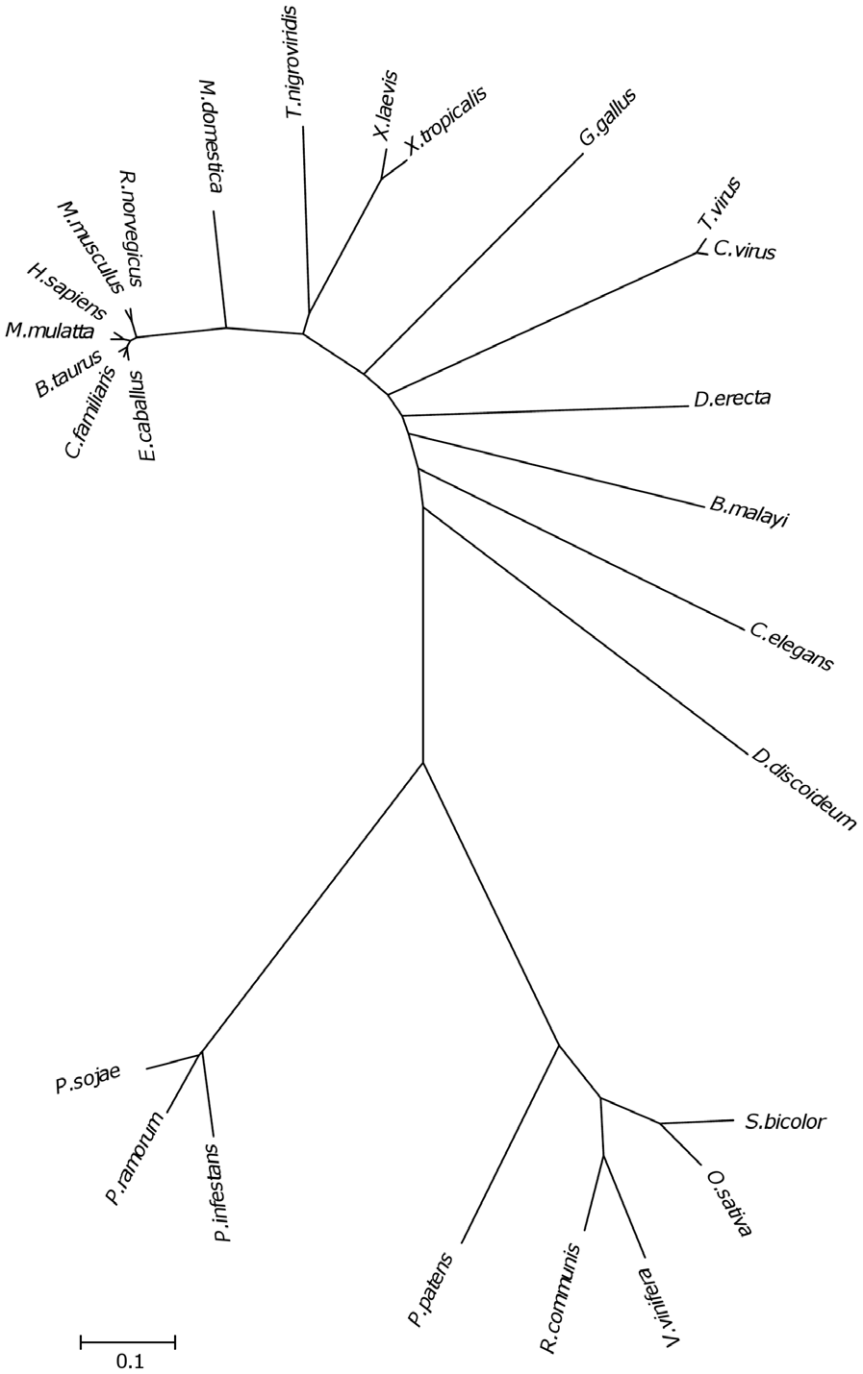
Phylogram of *Phytophthora* sPLD-like-A orthologs and their homologs in various organisms. The consensus minimal evolution tree constructed from the amino acid sequences is shown. For protein sequence identifiers see Table 3.

**Figure 4:**
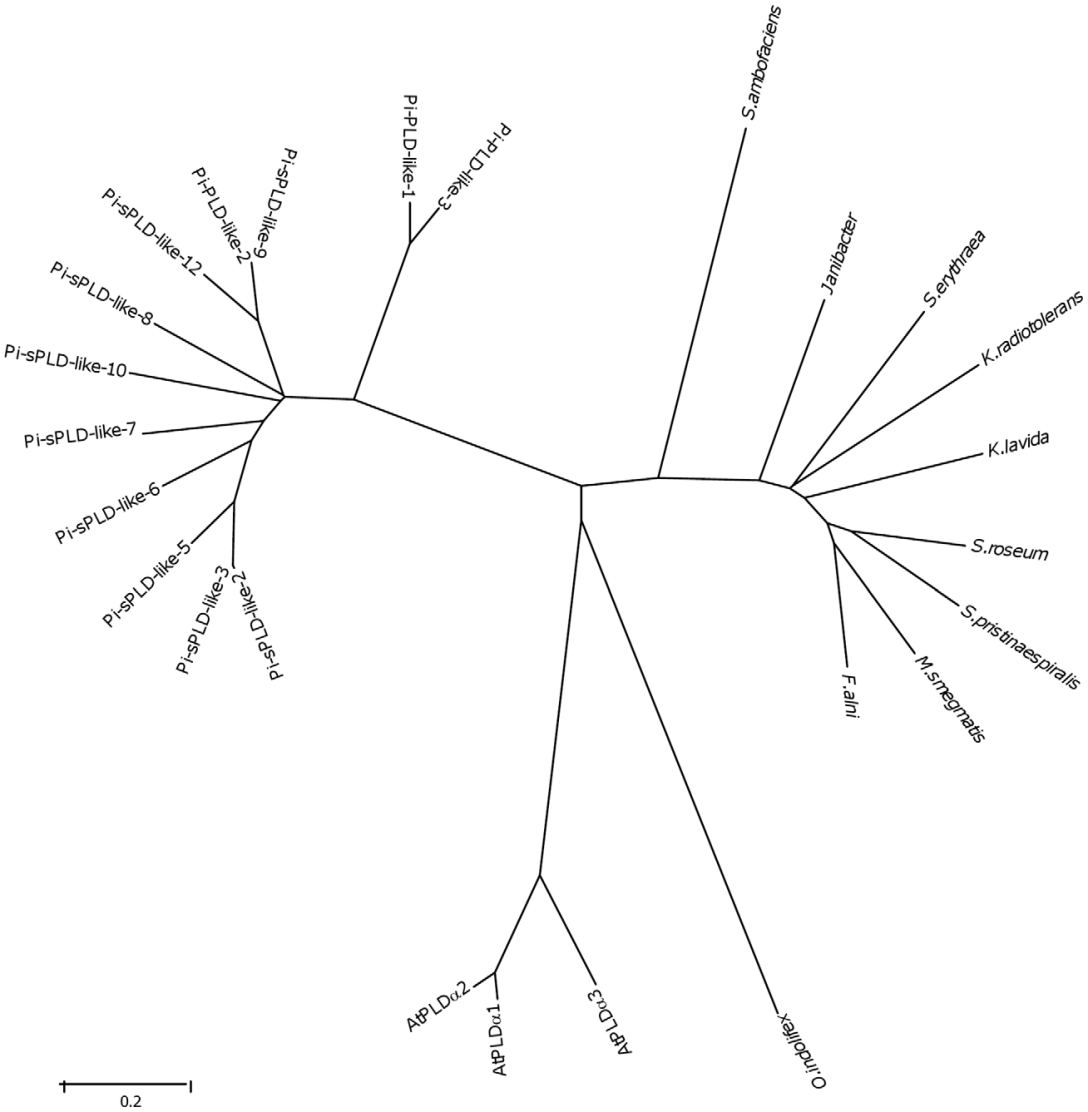
Alignment of amino acid sequences of type A sPLD-likes. Sequences are from *Phytophthora* (*P. infestans* sPLD-like-1), Grape (XP_002285518), Human (NP_001026866), *Drosophila* (XP_001974062) and Taterapox (YP_717345). The symbols used are [*] for identical, [;] for conservative and [.] for semi-conservative amino acid residues. Signal peptides/signal anchors are in italics, HKD motifs in bold and the “IGSANIN” motif is underlined.

**Figure 5:**
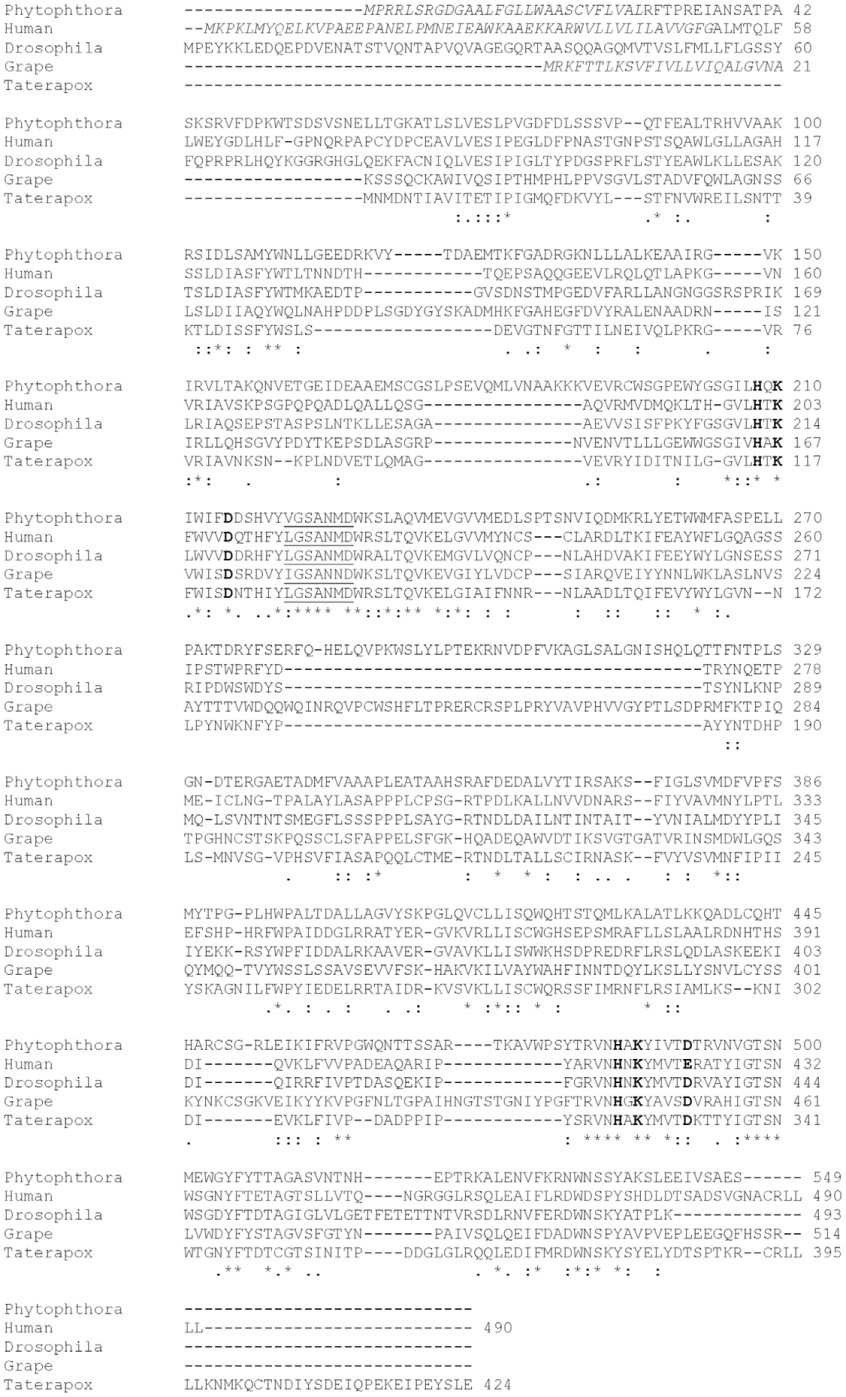
Phylogram of *P. infestans* PLD-likes and type B sPLD-likes and homologs in various organisms. The consensus minimal evolution tree constructed from the amino acid sequences is shown. For accession numbers of the *P. infestans* sequences see Table 1. Other sequences that were all selected by Blast searches are from: Arabidopsis (accession nr. NP_188194 for AtPLDα1, NP_175666 for AtPLDα2 and NP_197919 for AtPLDα3), *Frankia alni* (P_711983), *Janibacter* (ZP_00995229), *Kineococcus radiotolerans* (YP_001361943), *Kribbella flavida* (YP_003383471), *Mycobacterium smegmatis* (YP_884788), *Oceanibulbus indolifex* (ZP_02154842), *Saccharopolyspora erythraea* (YP_001106428), *Streptomyces ambofaciens* (CAJ89461), *Streptomyces pristinaespiralis* (YP_002197876) and *Streptosporangium roseum* (YP_003339462).

*Phytophthora* sPLD-like-A is quite divergent from other PLDs. The closest relatives are found in plants while more distant sPLD-like-A sequences are widespread in metazoan, viruses and prokaryotes. This suggests they are all descendants of an ancient gene (Figure 3; Table 3). In contrast, the sPLD-likes-Bs and PLD-likes are most analogous to PLD-like sequences derived from actinomycetes (Figure 4). Representative sPLD-like-A homologs were further analysed for potential characteristics (Table 3) and some clade representatives were aligned (Figure 5). It was noted that the prediction of the SP for *Phytophthora* orthologs is indecisive and hardly discriminates between a SP or a signal anchor (SA). Signal anchors are found in proteins that are inserted but not cleaved in the ER. sPLD-like-A homologs from plants and *Dictyostelium* have an obvious SP with an HMM score higher than 0.98. In contrast, a SA was predicted for many other sPLD-like-As, including all mammalian derived homologs (HMM score >0.98). Some, including those of *Caenorhabditis elegans, Drosophila erecta* and virus derived sequences, have neither a SP nor a SA (Table 3).

**Table 3:** Characteristics of sPLD-like-A homologs.

				N-terminus		Cleavage			
Organism	NCBI Nr.	BlastP	Protein	SP	SA	position	HKD1	HKD2	VGSANMD
*P. infestans*	EEY67671	-	549	0.48	0.52	28/29	HKD	HKD	VGSANMD
*P. sojae*	-	0.0	560	0.68	0.32	43/44	HKD	HKD	VGSANMD
*P. ramorum*	-	0.0	550	0.17	0.83	31/32	HKD	HKD	VGSANMD
*Ricinus communis*	XP_002515425	5E-55	516	0.93	0.07	28/29	HKD	HKD	IGSANND
*Oryza sativa*	NP_001058220	2E-53	512	1	0	28/29	HKD	HKD	IGSANND
*Physcomitrella patens*	XP_001769226	2E-52	511	1	0	17/18	HKN	HKD	LGSANND
*Sorghum bicolor*	XP_002438759	8E-52	516	1	0	28/29	HKD	HKD	IGSANND
*Vitis vinifera*	XP_002285518	4E-54	514	0.98	0.02	24/25	HKD	HKD	IGSANND
*Dictyostelium discoideum*	XP_637114	2E-42	438	0.96	0	19/20	HKD	HKE	VGSANAD
*Drosophila erecta*	XP_001974062	1E-44	493	0	0	-	HKD	HKD	LGSANMD
*Caenorhabditis elegans*	NP_504824	1E-40	516	0	0	-	HKD	HKD	IGSANMD
*Brugia malayi*	XP_001902241	3E-51	432	0	0	-	HKD	HKD	LGSANLD
*Taterapox virus*	YP_717345	6E-38	424	0	0	-	HKD	HKD	LGSANMD
*Cowpox virus*	ABD97389	1E-38	424	0	0	-	HKD	HKD	LGSANMD
*Xenopus laevis*	NP_001083260	2E-40	493	0	1	52/53	HKD	HKD	IGSANMD
*Xenopus tropicalis*	NP_001011023	4E-39	494	0	1	52/53	HKD	HKD	IGSANMD
*Tetraodon nigroviridis*	CAG12726	6E-34	493	0	0.99	49/50	HKD	HKD	IGSANMD
*Gallus gallus*	XP_421399	1E-33	517	0.01	0.51	43/44	HKD	HKD	IGSANMD
*Monodelphis domesticus* *	XP_001371155	2E-44	486	0	0.98	47/48	HKD	HKD	IGSANMD
*Mus musculus*	O35405	1E-43	488	0	1	51/52	HKD	HKE	LGSANMD
*Rattus norvegicus*	NP_001012167	4E-43	488	0	1	51/52	HKD	HKE	LGSANMD
*Macaca mulatta*	XP_001093926	2E-43	492	0	1	51/52	HKD	HKE	LGSANMD
*Homo sapiens*	NP_001026866	5E-42	490	0	1	51/52	HKD	HKE	LGSANMD
*Bos Taurus*	Q2KJJ8	4E-44	490	0	1	51/52	HKD	HKE	LGSANMD
*Equus caballus*	XP_001498901	1E-43	490	0	1	51/52	HKD	HKE	LGSANMD
*Canis familiaris*	XP_853110	2E-42	490	0	1	51/52	HKD	HKE	IGSANMD

*Based on alignments, Met84 was taken as protein start.

All sPLD-likes-As have a correct HKD1 except for *Physcomitrella patens.* For HKD2, a substitution is observed in nearly all mammalian and in the *Dictyostelium* homolog. The “VGSANMD” motif adjacent to the HKD1 motif is well conserved among all. *Phytophthora* sPLD-like-As are highly homologous (>75%) but with sPLD-like-As from other organisms the homology is much lower (Figure 5). The conserved regions mainly cover the HKD1 and HKD2 motifs and surrounding regions.

### Extracellular PLD activity

**Figure 6:**
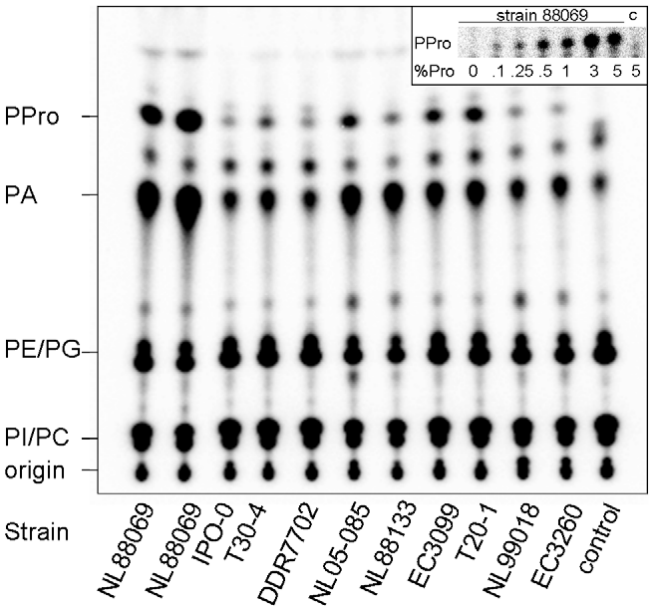
Profiles of plant phospholipids exposed to *P. infestans* extracellular medium reveals PLD activity. Metabotically labelled phospholipids were isolated from tobacco suspension cell culture, sonicated into vesicles, mixed with extracelllular medium collected from various *P. infestans* strains and incubated for 60 min in the presence of a primary alcohol (2% propanol). Lipids were (re)extracted, separated by TLC and visualized by phosphoimaging. As control, vesicles were treated with V8 medium. The origin, phosphatidylinositol (PI), phosphatidylcholine (PC), phosphatidylethanolamine (PE), phosphatidylglycerol (PG), phosphatidic acid (PA) and phosphatidylpropanol (PPro) are indicated. Inset, control medium (c) or extracellular medium (strain 88069) were incubated in the presence of propanol. %Pro indicates the final propanol concentration.

The finding that PLDs in two of the subfamilies have putative signal peptides urged us to validate if *P. infestans* secretes PLDs. First, the expression of the various PLD genes was analysed using *P. infestans* EST depositories [13] and Nimblegen data [14]. ESTs were identified (Table 1) for PXPH-PLD, PXTM-PLD, TM-PLD, sPLD-like-1, sPLD-like-12 and PLD-like-1. Most ESTs are derived from mycelial libraries which is in agreement with the Nimblegen expression profiles (Figure S1). The mycelial tissue was therefore tested as the source for extracellular PLD activity. A mix of metabolically labelled plant phospholipids was presented as substrate to cell free *P. infestans* extracellular medium. The reaction was performed in the presence of a primary alcohol. PLDs have the unique capability to transfer a phosphatidate intermediate to short-chain primary alcohols (acting as substitute for water) resulting in the formation of phosphatidylalcohols [15]. These transphosphatidylation products are easily detectable by thin layer chromatography (TLC) due to their metabolic stability and unique migration properties. Incubation of extracellular medium of *P. infestans* mycelial cultures with the metabolically labelled phospholipids in the presence of 2% propanol resulted in the production of both PA and phosphatidylpropanol (PPro) whereas in the control V8 medium no PLD activity was detected. Increase in PA and appearance of the non-pre-existing PPro was observed for all *P. infestans* strains tested (Figure 6) and this points to a ubiquitous presence of extracellular PLD activity. PA and PPro levels varied per strain which probably reflects differences in growth rate, viability of the mycelium, or variation in the amount of secreted activity among strains. Transphosphatidylation was already observed at the lowest propanol concentration tested (0.1%; Figure 6, inset).

To exclude that release of intracellular PLDs from dead or dying cells causes the observed activity, V8 medium of a flooded plate was carefully removed, fresh medium was added and after overnight incubation the extracellular medium was again tested for PLD activity. In the “fresh” extracellular medium PLD activity appeared to be even higher than in the extracellular medium prior to the replacement, demonstrating that the extracellular PLD activity is due to active release of PLD by living cells (Figure S2).

## Discussion

Eighteen PLD genes were identified in the genome of *P. infestans* and this correlates with findings in *P. sojae* and *P. ramorum* [4]. It clearly exceeds the number of PLD genes in yeasts, mammalians, and even plants. Also the number of PLD subfamilies in *Phytophthora* is staggering: six subfamilies including two distinct potentially secreted sPLD-like subfamilies, type A and B. Like many plant pathogens *Phytophthora* species have large secretomes [14], anticipating potential functions in pathogenicity. Our finding that *P. infestans* secretes PLD activity in the medium suggests that *Phytophthora* exploits PLDs to modify host tissues and as such, PLDs may function in pathogenicity.

*Phytophthora* PXPH-PLD and PXTM-PLD are closely related to canonical PXPH-PLDs as illustrated by conserved catalytic and regulatory domains with the exception of the transmembrane domains in PXTM-PLD. A preliminary functional analysis revealed that homozygous PXTM-PLD knock-out mutants show aberrant growth behaviour [16]. TM-PLD lacks homology with any other protein outside *Phytophthora*. Within *Phytophthora* species TM-PLD is highly conserved suggesting that this protein has a function in these organisms although not necessarily as a PLD.

The sPLD-like-Bs and PLD-likes have all regulatory domains typical for PLDs but lack the lipid binding domains and the DRY motif. Most possess an altered HKD1 motif lacking aspartate. Nevertheless, the motif might still be able to participate in a catalytic reaction. Attempts to express the genes in *E. coli* were unsuccessful. The transformation rate was low and in all viable transformants the constructs showed frameshifts or point mutations leading to inactive proteins (unpublished observations). The abundance of sPLD-like-B isoforms suggests that they have a prominent function.

In contrast to eleven sPLD-like-Bs, *P. infestans* has only one sPLD-like-A which, unlike sPLD-like-Bs, has an inconclusive prediction for its signal peptide. sPLD-like-A homologs exist outside oomycetes. The homology, however, is mostly restricted to the catalytic motifs and there is no uniform signal peptide prediction. The latter could point to different destinations of the various sPLD-like-As, in or outside the cell. Unlike the metazoan homologs, the plant homologs have an obvious signal peptide. This might be essential to cross cell walls and reach membranes in adjacent cells. Like plants, *Phytophthora* has cell walls so for *Phytophthora* sPLD-like-A one could argue in favour of a signal peptide for rather than a signal anchor. However, this needs further experimentation.

The large number of sPLD-likes suggests that *Phytophthora* secretes PLD activity. Since most sPLD-likes have a modified HKD motif, the substrate specificity is unknown and unanticipated activities could be encountered. Potential PLD activity monitoring problems, due to substrate specificity, phospholipid composition and ratio, alternative phospholipase and kinase activities and buffer preferences were circumvented by exploring a novel *in vitro* approach based on labelled plant phospholipids. We demonstrated that all tested *P. infestans* strains secrete PLD activity although the identity of the active enzyme remains to be established. The observed differences in PLD activity among strains probably reflects variations in growth rate, viability and the amount of enzyme(s) secreted. Analysis of similar samples by an alkaline TLC system revealed that PtdCho and PtdGro are the main substrates (data not shown). The PLD activity was capable to transphosphatidylate at low alcohol concentrations as expected for common PLDs [2]. This novel *in vitro* assay could be a first-class tool to dissect the biochemical characteristics of the PLD activity and to identify the enzyme(s) responsible.

*Phytophthora* is a hemibiotrophic plant pathogen and as such it is conceivable that secreted PLDs are utilized to function as generators of PA at the outside layer of host cell plasma membrane. PA has been characterized as a multifunctional phospholipid with direct or indirect impact on many cellular processes [1], [17]. In mammalian cells, exogenous added PLD activates G-protein-coupled receptors thereby mimicking hormones and growth factors [1]. Adding exogenous PA to *P. infestans* zoospores triggers encystment [9] whereas exogenous PA addition to plant cell-suspension culture results in MAPK activation, actin cytoskeleton rearrangements, reactive oxygen species generation, chlorosis, cell death responses [1], [8] and stimulation of secretion [17]. Ergo, if *Phytophthora* PLDs initiate PA production at the plant membrane several cellular processes may be activated that influence the infection process.

## Supporting Information

Figure S1 **Nimblegen microarray data for** ***Phytophthora infestans*** **PLD genes.** Each bar represents the average of two individual hybridisations. Samples were taken from *P. infestans* mycelium (strain T30-4) on various agar media (Pea agar, V8 agar and RS agar) or from infected potato leaves, 2–5 days post-inoculation (DPI). Nimblegen microarray data are available in GEO under accession number GSE14480 [14].
(TIF)

Figure S2 **PLD activity is continuously released into fresh medium.** Metabolically labeled phospholipids were incubated for 60 min in the presence of 2% propanol with fresh control medium (c), extracellular medium obtained by flooding (1^e^) and refreshed extracellular medium (2^e^) of *P. infestans* strain 88069. Lipids were extracted, separated by TLC and visualized by phosphoimaging.
(TIF)

Table S1 Amino acid identity between *P. infestans* sPLD-likes. The comparison was performed using Vector NTI and the results are presented as percentage (%) identity.
(DOC)
