# Correction: *Phytophthora infestans* Has a Plethora of Phospholipase D Enzymes Including a Subclass That Has Extracellular Activity

**DOI:** 10.1371/annotation/3355db50-d82f-4676-9c00-ab6c30ef7ee3

**Published:** 2011-03-24

**Authors:** Harold J. G. Meijer, Hussen Harrun Hassen, Francine Govers

The legends for figures 4 and 5 are switched. The correct legend for figure 4 is: "Phylogram of P. infestans PLD-likes and type B sPLD-likes and homologs in various organisms. The consensus minimal evolution tree constructed from the amino acid sequences is shown. For accession numbers of the P. infestans sequences see Table 1. Other sequences that were all selected by Blast searches are from: Arabidopsis (accession nr. NP_188194 for AtPLDa1, NP_175666 for AtPLDa2 and NP_197919 for AtPLDa3), Frankia alni (P_711983), Janibacter (ZP_00995229), Kineococcus radiotolerans (YP_001361943), Kribbella flavida (YP_003383471), Mycobacterium smegmatis (YP_884788), Oceanibulbus indolifex (ZP_02154842), Saccharopolyspora erythraea (YP_001106428), Streptomyces ambofaciens (CAJ89461), Streptomyces pristinaespiralis (YP_002197876) and Streptosporangium roseum (YP_003339462)." The correct legend for figure 5 is: "Alignment of amino acid sequences of type A sPLD-likes. Sequences are from Phytophthora (P. infestans sPLD-like-1), Grape (XP_002285518), Human (NP_001026866), Drosophila (XP_001974062) and Taterapox (YP_717345). The symbols used are [*] for identical, [;] for conservative and [.] for semi-conservative amino acid residues. Signal peptides/signal anchors are in italics, HKD motifs in bold and the "IGSANIN" motif is underlined."

