# Supplementary figures and images for: Phytophthora infestans Has a Plethora of Phospholipase D Enzymes Including a Subclass That Has Extracellular Activity
Source: PLoS One. 2011 Mar 14;6(3):e17767. doi: 10.1371/journal.pone.0017767 (PMC3056787; doi:10.1371/journal.pone.0017767)

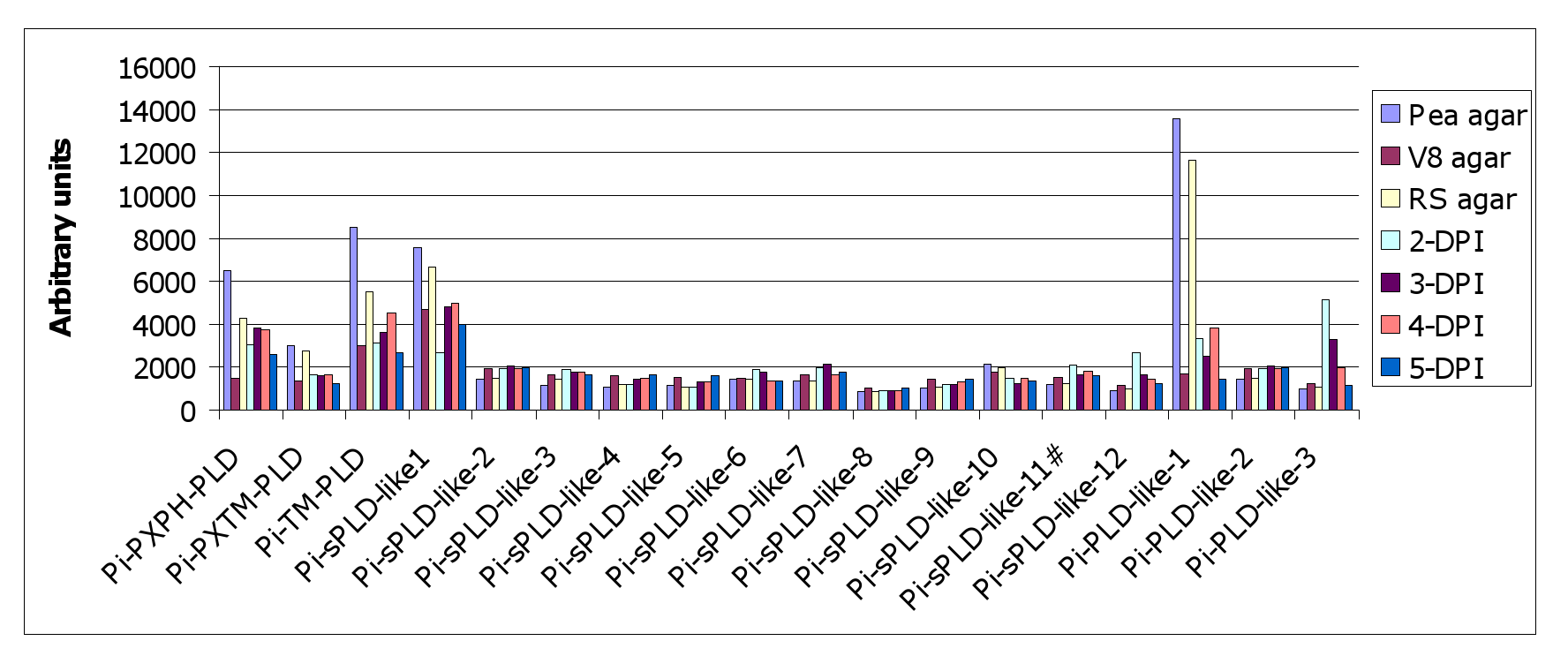

Supplement: Figure S1 — Nimblegen microarray data for Phytophthora infestans PLD genes. Each bar represents the average of two individual hybridisations. Samples were taken from P. infestans mycelium (strain T30-4) on various agar media (Pea agar, V8 agar and RS agar) or from infected potato leaves, 2–5 days post-inoculation (DPI). Nimblegen microarray data are available in GEO under accession number GSE14480 [14]. (TIF) [file pone.0017767.s001.tif]

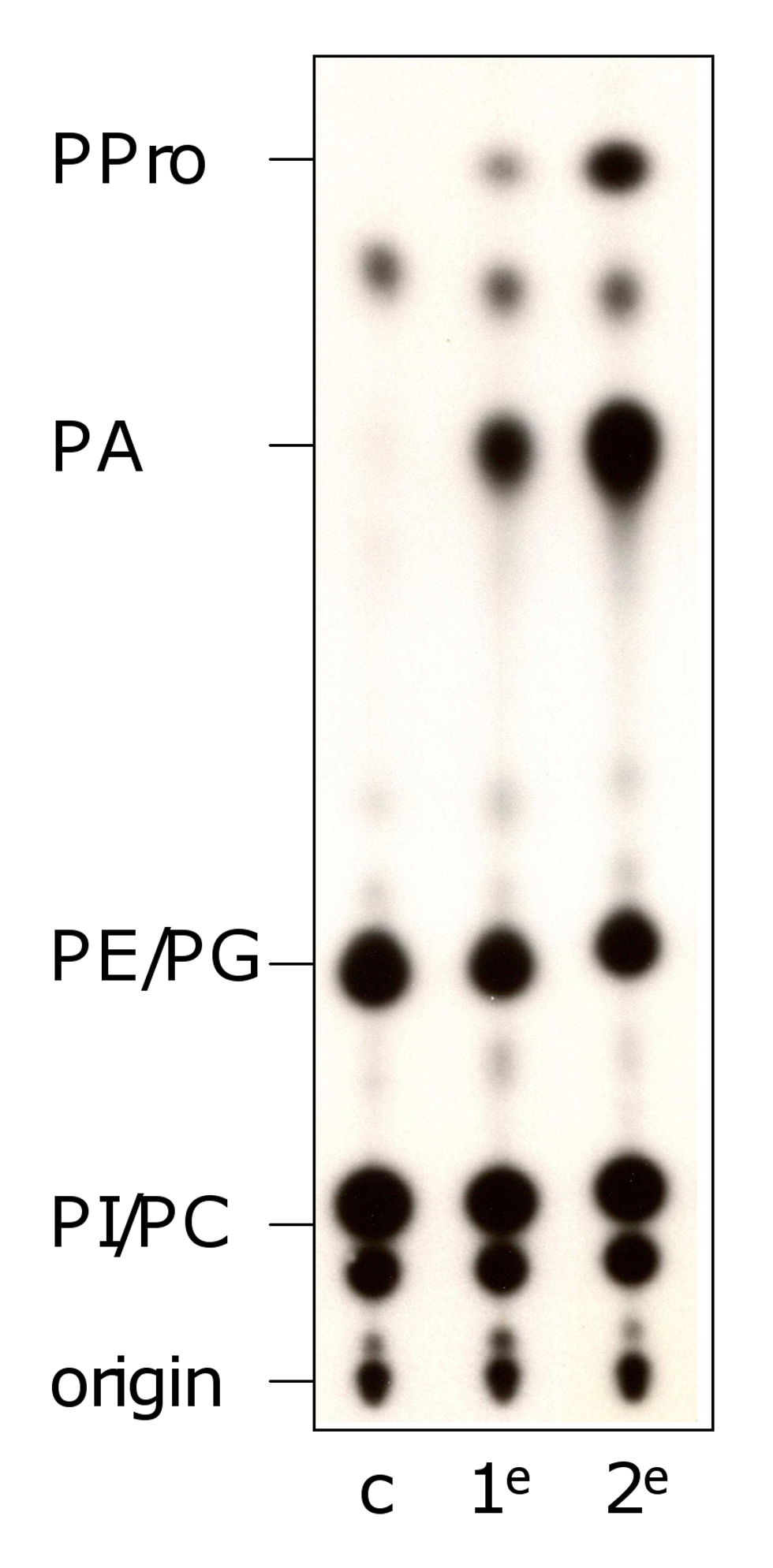

Supplement: Figure S2 — PLD activity is continuously released into fresh medium. Metabolically labeled phospholipids were incubated for 60 min in the presence of 2% propanol with fresh control medium (c), extracellular medium obtained by flooding (1e) and refreshed extracellular medium (2e) of P. infestans strain 88069. Lipids were extracted, separated by TLC and visualized by phosphoimaging. (TIF) [file pone.0017767.s002.tif]
