# Supplementary material for: Phytophthora infestans Has a Plethora of Phospholipase D Enzymes Including a Subclass That Has Extracellular Activity
Source: PLoS One. 2011 Mar 14;6(3):e17767. doi: 10.1371/journal.pone.0017767 (PMC3056787; doi:10.1371/journal.pone.0017767)
Supplement: Table S1 — Amino acid identity between P. infestans sPLD-likes. The comparison was performed using Vector NTI and the results are presented as percentage (%) identity. (DOC) [file pone.0017767.s003.doc]

**Table S1. Amino acid identity between *P. infestans* sPLD-likes.**

| sPLD-like Nr. | 1 | 2 | 3 | 4 | 5 | 6 | 7 | 8 | 9 | 10 | 11 | 12 | sPLD-like |
| --- | --- | --- | --- | --- | --- | --- | --- | --- | --- | --- | --- | --- | --- |
| 1 |  | 11 | 11 | 12 | 12 | 12 | 13 | 14 | 12 | 12 | 13 | 13 | Type A |
| 2 |  |  | 99 | 76 | 76 | 65 | 61 | 58 | 56 | 56 | 53 | 54 | Type B |
| 3 |  |  |  | 76 | 76 | 65 | 61 | 58 | 56 | 56 | 54 | 54 | Type B |
| 4 |  |  |  |  | 98 | 66 | 62 | 56 | 55 | 56 | 54 | 54 | Type B |
| 5 |  |  |  |  |  | 66 | 62 | 56 | 55 | 57 | 54 | 54 | Type B |
| 6 |  |  |  |  |  |  | 59 | 57 | 55 | 57 | 54 | 53 | Type B |
| 7 |  |  |  |  |  |  |  | 54 | 56 | 57 | 55 | 54 | Type B |
| 8 |  |  |  |  |  |  |  |  | 57 | 58 | 56 | 56 | Type B |
| 9 |  |  |  |  |  |  |  |  |  | 55 | 76 | 76 | Type B |
| 10 |  |  |  |  |  |  |  |  |  |  | 54 | 55 | Type B |
| 11 |  |  |  |  |  |  |  |  |  |  |  | 94 | Type B |
| 12 |  |  |  |  |  |  |  |  |  |  |  |  | Type B |

The comparison was performed using Vector NTI and the results are presented as percentage (%) identity.
